# Preselecting Variants from Large-Scale Genome-Wide Association Study Meta-Analyses Increases the Genomic Prediction Accuracy of Growth and Carcass Traits in Large White Pigs

**DOI:** 10.3390/ani13243746

**Published:** 2023-12-05

**Authors:** Chen Wei, Chengjie Chang, Wenjing Zhang, Duanyang Ren, Xiaodian Cai, Tianru Zhou, Shaolei Shi, Xibo Wu, Jinglei Si, Xiaolong Yuan, Jiaqi Li, Zhe Zhang

**Affiliations:** 1 National Engineering Research Centre for Swine Breeding Industry, Provincial Key Laboratory of Agricultural Animal Genomics and Molecular Breeding, College of Animal Science, South China Agricultural University, Guangzhou 510640, China; weichenwjf@126.com (C.W.); komorebijc@163.com (C.C.); chinj_cheung@163.com (W.Z.); ren457842071@163.com (D.R.); cxt0804@163.com (X.C.); tianruzhou516@163.com (T.Z.); shisl8079@163.com (S.S.); yxl@scau.edu.cn (X.Y.); jqli@scau.edu.cn (J.L.); 2 Guangxi State Farms Yongxin Animal Husbandry Group Co., Ltd., Nanning 530022, China; wuxibo@126.com (X.W.); jinglei7139@126.com (J.S.)

**Keywords:** genomic prediction, preselected variants, large-scale genome-wide association studies meta-analysis, growth and carcass traits, pig

## Abstract

**Simple Summary:**

Preselected potential causal variants from genome-wide association studies (GWASs) for genomic prediction is an appealing strategy for improving the accuracy of genomic prediction. Here, we performed genomic prediction using preselected variants identified with a large GWAS meta-analysis on three growth and carcass traits in Large White pigs. Compared to using the SNP chip data, the accuracies of genomic predictions based on different SNP preselection strategies were on average improved by 0.3 to 2% using the genomic best linear unbiased prediction given genetic architecture (BLUP|GA) model. Our results provide new opportunities for future applications of genomic prediction in livestock and poultry by using large amounts of genotype data.

**Abstract:**

Preselected variants associated with the trait of interest from genome-wide association studies (GWASs) are available to improve genomic prediction in pigs. The objectives of this study were to use preselected variants from a large GWAS meta-analysis to assess the impact of single-nucleotide polymorphism (SNP) preselection strategies on genome prediction of growth and carcass traits in pigs. We genotyped 1018 Large White pigs using medium (50k) SNP arrays and then imputed SNPs to sequence level by utilizing a reference panel of 1602 whole-genome sequencing samples. We tested the effects of different proportions of selected top SNPs across different SNP preselection strategies on genomic prediction. Finally, we compared the prediction accuracies by employing genomic best linear unbiased prediction (GBLUP), genomic feature BLUP and three weighted GBLUP models. SNP preselection strategies showed an average improvement in accuracy ranging from 0.3 to 2% in comparison to the SNP chip data. The accuracy of genomic prediction exhibited a pattern of initial increase followed by decrease, or continuous decrease across various SNP preselection strategies, as the proportion of selected top SNPs increased. The highest level of prediction accuracy was observed when utilizing 1 or 5% of top SNPs. Compared with the GBLUP model, the utilization of estimated marker effects from a GWAS meta-analysis as SNP weights in the BLUP|GA model improved the accuracy of genomic prediction in different SNP preselection strategies. The new SNP preselection strategies gained from this study bring opportunities for genomic prediction in limited-size populations in pigs.

## 1. Introduction

Pork is one of the most important sources of animal protein for humans [1]. At present, growth and carcass traits such as average daily gain at 100 kg (ADG100), average back fat thickness at 100 kg (BFT100), and days to 100 kg (DAYS100) are recognized as the most important breeding objectives in pig breeding programs due to their considerable influence on pork production and quality [2,3,4]. The genetic improvement of these traits represents a compelling avenue for augmenting pork production and quality. With advances in genotyping technologies, genomic selection (GS) or genomic prediction, proposed by Meuwissen et al. [5], has been introduced and applied to address the limitations of traditional breeding methods in livestock populations, particularly in terms of improving prediction accuracy of traits and decreasing generation intervals [6].

Presently, GS has been widely applied in modern pig breeding. Since its early implementations, genomic prediction has typically been performed by using SNP chip data that capture the effects of the causal variants (often unknown) through LD, which can modestly increase the accuracy of genomic predictions for economic traits of pigs [7,8,9]. In contrast, genomic prediction using whole-genome-sequence (WGS) data is expected to enhance the accuracy of genomic predictions. This is achieved by incorporating causal variants or SNPs in strong LD with causal variants that influence the target traits. Simulation studies have substantiated that the utilization of WGS data, as opposed to SNP chip data, engenders a noteworthy enhancement in predictive accuracy. Specifically, the improvements observed are in the range of 2.5 to 5.9% within populations [10,11] and a considerably more substantial range of 22 to 31% for multi-breed populations [11]. Another simulation study showed that the accuracy of genomic prediction increased by up to 30% when WGS data captured causal variants with a low minor-allele frequency [12]. However, in practical applications, the use of WGS data for genomic prediction in pig populations has not shown substantial improvements in accuracy compared to traditional SNP chip data, particularly when analyzed within specific breeds [13,14]. Other studies have found a slight increase in prediction accuracy (e.g., from 0.3 to 3.5%) for some economic traits within or across pig populations, depending on the different prediction methods used [15,16]. Nevertheless, the suboptimal outcomes observed in genomic prediction when employing WGS data can be ascribed to the presence of a considerable surplus of redundant SNPs. These neighboring SNPs are probably in strong LD with causative variants or with other SNPs located in specific genomic blocks [17,18].

One of the most successful strategies to improve the accuracy of genomic predictions is the utilization of preselected variants associated with the trait of interest from WGS data. In recent years, preselected potential causal variants identified through genome-wide association studies (GWASs) have already been applied in genomic prediction to improve the prediction accuracy for a variety of traits in livestock and poultry [19,20], including pigs [21,22]. In certain instances, this strategy has resulted in improved accuracy of genomic prediction for growth and carcass traits in pigs, with improvements ranging from 0.9 to 46% for multi-breed populations [21,22,23]. However, it should be noted that this strategy did not result in improved prediction accuracy in all cases [21,24,25]. Fine mapping of causal variants was still challenging, and the advantages for genomic predictions were limited.

A simulation study indicated that the detection of causal variants explained only 20% of the total genetic variance when using a sample size of 7000 for a GWAS in an effective population size of 20 [23]. Recently, Ros-Freixedes et al. [21] used preselected SNPs based on GWAS results for genomic prediction, studying a population of nearly 100,000 pigs from the largest lines with imputed WGS data. Compared to the use of SNP chips, they found marginal or no gains (from −3.4 to 9.8%) in prediction accuracy for eight common complex traits in pigs. In these data, the preselected SNP panels consisted of standard chip data augmented with causal variants from GWAS results or the top 40,000 variants with the lowest *p*-values from GWAS results. Subsequently, Jang et al. [26] also indicated marginal or no gains (from 0.1 to 4.9%) in prediction accuracy for eight common complex traits in pigs when using single-step genomic best linear unbiased prediction (ssGBLUP) with BayesR SNP variances as weights on the preselected SNP panels described above. However, these preselected SNPs may contain a number of redundant SNP or may have a small effect on the trait of interest, which could restrict the available information for genomic prediction.

Several studies have demonstrated the importance of using estimated marker effects from existing GWAS results, either within the dataset or from public sources. These marker effects can be used to construct weighted genomic relationship matrices (G) in the prediction model, resulting in improved accuracy of genomic predictions compared to traditional methods such as genomic best linear unbiased prediction (GBLUP), ridge regression BLUP (rr-BLUP), and BayesB [27,28]. However, another two studies reported that using estimated marker effects obtained from GWAS results as weighting factors to construct a weighted G matrix for genomic prediction resulted in lower accuracy compared to the Bayesian model [28,29]. A previous study reported that using the top SNPs selected according to their estimated marker effects obtained from the rr-BLUP model for genomic prediction results in superior accuracy of genomic prediction compared to WGS data [30]. This is mainly due to capturing the major genes that affect the trait, thereby improving the prediction ability of the G matrix. Therefore, using the top SNPs selected according to their estimated marker effects obtained from the rr-BLUP model for genomic prediction is expected to improve the accuracy of genomic prediction for growth and carcass traits in pigs.

To investigate the effect of leveraging preselected SNPs on genomic prediction, we used preselected variants associated with growth and carcass traits based on a large-scale GWAS meta-analysis containing over 40,000 pigs from nine populations of Large White pigs (http://pigbiobank.farmgtex.org, accessed on 1 April 2023). The objectives of this study were to use preselected variants from a large-scale GWAS meta-analysis: (1) to assess the impact of different SNP preselection strategies for genome prediction compared to using all variants from the SNP chip data; (2) to evaluate the impact of different proportions of top selected SNPs for genome prediction within different SNP preselection strategies; and (3) to identify the most efficient prediction models for a variety of traits within different SNP preselection strategies.

## 2. Materials and Methods

### 2.1. Animals and Phenotypes

In this study, a total of 1026 Large White pigs (194 boars and 832 sows) were used, originating from the breeding nucleus farm in Guangxi (Guangxi, Nanning, China), spanning from July 2017 to June 2021. The average age of the pigs was 186 days. In addition, all pigs were raised with the same diet and under identical conditions, and they were fed according to large-scale breeding conditions. Phenotypic observations included three growth and carcass traits: ADG100, BFT100, and DAYS100. The descriptive statistics for the growth and carcass traits analyzed in this work are listed in Table 1, along with the frequency distribution of the phenotypic data in Figure S1. The ADG100 trait was defined as the animal’s body weight measured at the off test divided by the animal’s age. The DAYS100 trait was collected when the pigs were taken off test and then adjusted to days at 100 kg body weight. The BFT100 trait was measured between the 10th and 11th rib of Large White pigs using an Aloka-SSD-500 B ultrasound device (Corometrics Medical Systems, USA) at the off test and then adjusted to the average back fat thickness at 100 kg body weight. We calculated the correction of ADG100, DAYS100, and BFT100 according to the formula described by the Swine Genetic Prediction Project (http://www.breeding.cn, accessed on 12 December 2022) [31]. Phenotypes were then corrected for removing the identified fixed effects by using the lm () function in base R. The formula is as follows:

(1) $$Y_{ijkmn}=\mu +{Year}_{i}+{Season}_{j}+{Sex}_{k}+{Farm}_{m}+e_{ijkmn}\;$$

where $Y_{ijkmn}$ represents the measured trait value of the mth individual; μ is the general mean; ${Year}_{i}$ represents the fixed effect of the year of the start of testing (*i* = 2017–2021); ${Season}_{j}$ represents the fixed effect of the season of the start of testing (*j* = 1~2; 1: May–October, and 2: January–April and November–December); ${Sex}_{k}$ represents the fixed effect of sex (*k* =1~2; 1: boar, and 2: sow); ${Farm}_{m}$ represents the fixed effect of farm at the start of testing (*m* = 11); $e_{ijkmn}$ represents the random residual effect.

### 2.2. Genotyping and Imputation

In our study, 1018 Large White pigs were genotyped by using the Geneseek GGP50k (Neogen Corporation, Lansing, MI, USA), comprising 45,073 SNPs. We filtered out variants with an SNP call rate < 0.05, MAF < 0.05, and the Hardy–Weinberg equilibrium < 1 × 10^−5^. Finally, a total of 38,678 SNPs in the study population were kept for further study. We then imputed the filtered SNPs on autosomes to sequence level with Beagle (version 5.1) [32] using 1602 WGS samples from the PigGTEx PGRP (http://pigbiobank.farmgtex.org, accessed on 1 April 2023) as a reference panel. To evaluate the accuracy of genotype imputation from the SNP chip, we used a 20-fold cross-validation method that was orderly masked for 5% of the SNP genotypes of all animals, and all the SNP chips were then imputed using Beagle (version 5.1). Finally, the imputation accuracy was evaluated as 0.952 based on Pearson’s correlation between the imputed and true genotypes. After imputation, we filtered out variants with an SNP call rate < 0.05, minor-allele frequency < 0.05, the Hardy–Weinberg equilibrium < 1 × 10^−5^, and LD > 0.8 (50 kb) by using PLINK software (version 1.9) [33]. Finally, a total of 863504 SNPs in the study population were retained for further study.

### 2.3. Variant Selection Based on Genome-Wide Association Meta-Analysis

To investigate the impact of preselected variants on genomic prediction, we obtained the summary statistics of large GWAS meta-analyses from the PigBiobank, which is part of the Pig Genotype-Tissue Expression (PigGTEx) project. The summary statistics were downloaded from the website http://pigbiobank.farmgtex.org (accessed on 1 April 2023). These datasets included samples from nine populations, totaling 28,967 (ADG100), 45,863 (BFT100), and 37,618 (DAYS100) samples. For each phenotype, we finally obtained the GWAS meta-analysis information of 16,860,419~27,288,578 SNPs.

To test whether preselected variants from the GWAS summary statistics data could provide better prediction accuracy than all variants from the SNP chip data or WGS data, we conducted genomic prediction using all variants from the SNP chip data and the WGS data. The SNP chip data (also defined as ‘Chip’) were adopted as the benchmark set for prediction accuracy. We assessed different SNP preselection strategies for preselected variants based on GWAS meta-analysis results:

(1) Selected top SNPs based on *p*-value ranking: We first ranked the SNPs based on the *p*-value of the GWAS meta-analysis results, then matched SNPs from the SNP chip data or WGS data in the GS study to the GWAS meta-analysis results according to the SNP’s physical location. We therefore ranked the SNPs from the GS population and defined them as P_top_Chip and P_top_WGS strategies, respectively. For the P_top_Chip strategy, we preselected the top 1%, 5%, 10%, 20%, 40%, 60%, 80%, and 100% variants from the SNP chip data with the lowest *p*-value based on the GWAS meta-analysis summary statistics in the GS population. For the P_top_WGS strategy, we preselected the top 0.1%, 0.3%, 0.5%, 1%, 5%, 10%, 20%, 40%, 60%, 80%, and 100% variants from the WGS data with the lowest *p*-value based on the summary statistics of GWAS meta-analysis in the GS population. The number of selected top SNPs from the SNP chip data and the WGS data were presented in Table 2. In addition, we preselected the most significant SNPs (*p* < 0.01) from the GWAS results (i.e., 5% of the SNPs) to compare the prediction accuracy of all markers from the SNP chip data and other alternative strategies, as already proposed by de los Campos et al. [34].

(2) Selected top SNPs based on estimated marker effects of the trait: Initially, we conducted the rr-BLUP analysis using LDAK v5.0 [35] to estimate marker effects using either the SNP chip data or WGS data from the GS population. Subsequently, we matched SNPs from the SNP chip data or WGS data in the GS study to the GWAS meta-analysis results according to the SNP’s physical location. Then, we obtained the corresponding effect for these SNPs based on the rr-BLUP analysis results from the SNP chip data or WGS data in the GS population. Finally, we ranked the SNPs based on the absolute value of these estimated marker effects obtained from the SNP chip data or WGS data in the GS population and defined them as G_top_Chip and G_top_WGS strategies, respectively. For the G_top_Chip strategy, we preselected the top 1%, 5%, 10%, 20%, 40%, 60%, 80%, and 100% variants from the SNP chip data with the highest estimated effect size in the GS population. For the G_top_WGS strategy, we preselected the top 0.1%, 0.3%, 0.5%, 1%, 5%, 10%, 20%, 40%, 60%, 80%, and 100% variants from the WGS data with the highest estimated effect size in the GS population.

### 2.4. Genomic Prediction Models

In this study, we implemented five models to perform genomic prediction.

#### 2.4.1. GBLUP

We used GBLUP as the benchmark model to evaluate other models presented in this study. The model includes a single random genetic effect, and is as follows:

(2) Y=μ+Zg+e

where Y is a vector of corrected phenotypes for ADG100, BFT100, or DAYS100; μ is the general mean; Z is a matrix of variant genotypes; $g\sim N(0,{G\sigma }_{g}^{2})$ represents the additive genetic effects, where G and $\sigma_{g}^{2}$ denote the genomic relationship matrix [36] and additive genetic variance, respectively; $e\sim N(0,{I\sigma }_{e}^{2})$ represents the random residuals, where I and $\sigma_{e}^{2}$ denote the identity matrix and residual variance, respectively. The G matrix was constructed using all SNPs according to:

(3) $$G=\frac{ZZ^{\prime }}{2\sum_{i=1}^{m}p_{i}\left(1-p_{i}\right)}$$

where Z is a matrix of centered genotypes, with elements $\left(0-{2p}_{i}\right)$, $\left(1-{2p}_{i}\right)$, $\left(1-{2p}_{i}\right)$ denoting 11 homozygous, 12 or 21 heterozygous, and 22 homozygous, respectively; and $p_{i}$ is the minor-allele frequency of the *i*th SNP.

#### 2.4.2. GFBLUP

The genomic feature BLUP (GFBLUP) model is an extension of the linear mixed model used in general GBLUP, which includes two components [37]. The prediction model is as follows:

(4) Y=μ+Zf+Zg+e

where $f\sim N(0,{G_{f}\sigma }_{f}^{2})$ represents additive genetic effects captured by a proportion of top SNPs selected, where $G_{f}$ and $\sigma_{f}^{2}$ denote the genomic relationship matrix and additive genetic variance captured by the top SNPs, respectively; $g\sim N(0,{G\sigma }_{g}^{2})$ represents additive genetic effects captured by all genetic variants, where G and $\sigma_{g}^{2}$ denote the genomic relationship matrix [36] and additive genetic variance, respectively.

#### 2.4.3. TABLUP

The trait-specific relationship matrix BLUP (TABLUP) model is an improvement of the RRBLUP and GBLUP models; it emphasizes trait-specific SNPs to explain more of the genetic variance in the trait [30]. The statistical model can be expressed as follows:

(5) $$Y=\mu +ZS_{a}+e$$

where $S_{a}\sim N(0,{S_{a}\sigma }_{s_{a}}^{2})$ represents additive genetic effects captured by all SNPs, and $\sigma_{s_{a}}^{2}$ is the additive genetic variance captured by all SNPs. The $S_{a}$ matrix is defined as:

(6) $$S_{a}=\frac{MDM^{\prime }}{2\sum_{i=1}^{m}p_{i}\left(1-p_{i}\right)}$$

where M represents the genotype matrix of all SNPs; D is a diagonal matrix, and its diagonal elements diag (D) contain the weights as the estimated marker effects obtained from the GWAS meta-analysis results for the selected SNPs in M. Of the remaining SNPs, we rescaled diag (D) so that the mean of diag (D) was 1 to facilitate the interpretation of the model results.

#### 2.4.4. BLUP|GA

The BLUP|GA (BLUP approach conditional on the genetic architecture) model is an improvement of the TABLUP model [30]. This is achieved by constructing a trait-specific covariance matrix (T), which is a weighted sum of the $S_{f}$ matrix and the G matrix, and was calculated as follows:

(7) $$T=\omega S_{f}+\left(1-\omega \right)G$$

where ω is the overall weight for the $S_{f}$ matrix, which was set to 0.1, 0.3, 0.5, 0.7, and 0.9; the $S_{f}$ and G matrices are genomic relationship matrixes, which are calculated by the selected SNPs and all SNPs, respectively. The $S_{f}$ matrix is defined as:

(8) $$S_{f}=\frac{MDM^{\prime }}{2\sum_{i=1}^{m}p_{i}\left(1-p_{i}\right)}$$

where M represents the genotype matrix of selected top SNPs; D is a diagonal matrix, and its diagonal elements, diag (D), contain the weights as the estimated marker effect obtained from the GWAS meta-analysis results for the selected SNPs in M. To ensure the analogy between $S_{f}$ and *G*, we rescaled diag (D) so that the mean of diag (D) was 1 to facilitate interpretation of the model results.

#### 2.4.5. GTBLUP

The genomic trait-specific BLUP (GTBLUP) model utilizes estimated marker effects of selected SNPs to construct the weighted G, including two components similar to the GFBLUP model. The statistical model for GTBLUP can be expressed as:

(9) $$Y=\mu +Z_{1}g+Z_{2}s_{f}+e$$

where all variables are defined as described above.

#### 2.4.6. Evaluation of the Accuracy of GEBV

In this study, we estimated the accuracy of genomic prediction using the R-regress [38]. We then used a 5×tenfold cross-validation with replicate 50 times, resulting in 50 averaged accuracies of genomic prediction. The accuracy of genomic prediction was calculated as Pearson’s correlation coefficient of the corrected phenotype and GEBV, and was as follows:

(10) $$r_{accuracy}=\frac{Cov\left(X,Y\right)}{\sqrt{var\left(X\right)var\left(Y\right)}}$$

where ***X*** and ***Y*** represent the corrected phenotype and GEBV of all individuals, respectively; $Cov\left(X,Y\right)$ represents the covariance of ***X*** and ***Y***; $var\left(X\right)$ represents the variance of ***X***; and $var\left(Y\right)$ represents the variance of ***Y***.

## 3. Results

### 3.1. The Impact of Alternative Strategy for Preselecting Variants on Genome Prediction

**Figure 1:**
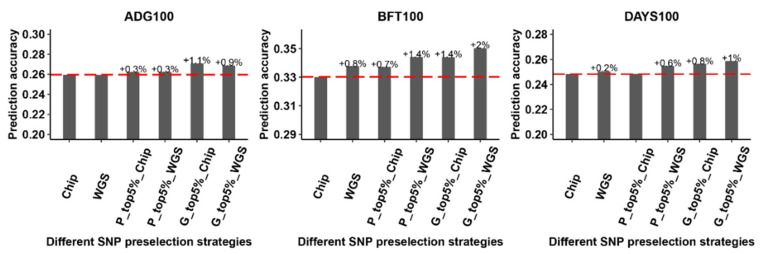
The impact of different alternative strategies for preselecting variants on genome prediction. “Chip” represents all variants from the SNP chip data, and “WGS” represents all variants from the WGS data. “P_top5%_Chip” and “P_top5%_WGS”represent the top 5% of SNPs based on *p*-value ranking from a large GWAS meta-analysis in the SNP chip and WGS data, respectively. “G_top5%_Chip” and “G_top5%_WGS” represent the top 5% of SNPs based on estimated marker effects from an rr-BLUP analysis in the SNP chip and WGS data, respectively. The dashed lines indicate the value of Chip as a reference. ADG100: average daily gain at 100 kg, BFT100: average back fat thickness at 100 kg, DAYS100: days to 100 kg.

To investigate the impact of preselected variants on genome prediction, we implemented four strategies to preselect variants from a large GWAS meta-analysis. We preselected the most significant SNPs (*p* < 0.01) from a GWAS meta-analysis (i.e., 5% of the SNPs) and used the BLUP|GA model with the best weight parameter (ω = 0.1) for genome prediction across different SNP preselection strategies (Table S1, Figures S2 and S3). Figure 1 and Table S2 show the prediction accuracy for the three traits using preselecting variants from the four different SNP preselection strategies and all variants from the SNP chip data and WGS data. Across all three traits, when compared to using all markers from the SNP chip data, we observed that the prediction accuracy increased by 0.3 to 0.7% for P_top5%_Chip, by 0.3 to 1.4% for P_top5%_WGS, by 0.8 to 1.1% for G_top5%_Chip, and by 0.9 to 2% for G_top5%_WGS. We also found that all markers from the WGS data increased by 0.2 to 0.8% or even did not change compared with all markers of the SNP chip data, indicating that further increasing the marker density does not improve prediction accuracy. In addition, we found that the prediction accuracy was slightly higher for preselecting variants from P_top5%_WGS, G_top5%_Chip, and G_top5%_WGS scenarios compared to using all markers of the WGS data, with values ranging from 0.3 to 1.2%.

### 3.2. The Impact of Different Proportion of Selected Top SNPs on Genome Prediction

**Figure 2:**
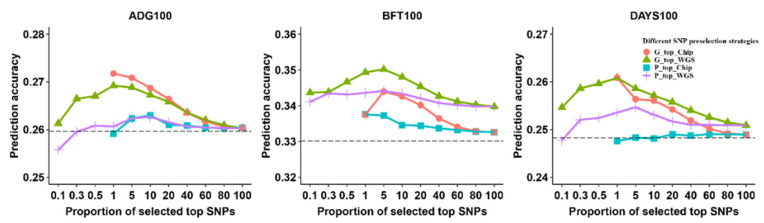
The impact of different proportions of selected top SNPs on genome prediction “P_top_Chip” and “P_top_WGS” represent top SNPs based on *p*-value ranking from a large GWAS meta-analysis in the SNP chip and WGS data, respectively. “G_top_Chip” and “G_top_WGS” represent top SNPs based on estimated marker effects from an rr-BLUP analysis in the SNP chip and WGS data, respectively. The dashed lines indicate the value of the SNP chip as a reference. ADG100: average daily gain at 100 kg, BFT100: average back fat thickness at 100 kg, DAYS100: days to 100 kg.

To assess the impact of different proportions of selected top SNPs on genome prediction, we categorized them into eight or eleven SNP categories. These categories were determined based on either the *p*-values obtained from a large GWAS meta-analysis or the estimated marker effects derived from an rr-BLUP analysis conducted in this study population. We used the BLUP|GA model with the best weight parameter (ω = 0.1) for genome prediction across different SNP preselection strategies (Table S1, Figures S2 and S3). Figure 2 and Table S3 show the prediction accuracy of three traits based on using different proportions of selected top SNPs for four SNP preselection strategies. We found a higher prediction accuracy using selected top SNPs for four strategies in all traits compared with all markers from the chip data, except for ADG100 using 0.1% of the top SNPs for P_top_WGS and 1% of the top SNPs for P_top_Chip. For the P_top_WGS and G_top_WGS strategies, we found that the prediction accuracy in three traits showed a trend of rising and then declining when the proportion of selected top SNPs increased. When using 0.1 to 0.5% of the top SNPs (containing from 854 to 4290 significant variants) for genome prediction, we observed a gradual improvement in prediction accuracy with an increasing number of variants impacting the trait. The highest prediction accuracy was observed when using 1% or 5% of the top SNPs, followed by a gradual decline when the proportion of selected top SNPs increased from 10 to 100%. This decrease in prediction accuracy might be attributed to the increase in noise caused by the inclusion of non-associated variants. For the G_top_Chip strategy, we found that prediction accuracy showed a trend of decline when using 1 to 100% of the top SNPs from the SNP chip data, with the highest prediction accuracy using 1% or 5% of the top SNPs. For the P_top_Chip strategy, we found that prediction accuracy showed a trend of decline (ADG100 and BFT100) or no change (DAYS100).

### 3.3. The Impact of Different Models of Selected Top SNPs on Genome Prediction

We compared the prediction performance of different prediction models, including the GBLUP, GFBLUP, BLUP|GA, GTBLUP, and TABLUP models. Using the prediction accuracy of GBLUP as the reference, the influences of different prediction models varied under different strategies for three traits based on different proportions of selected top SNPs (Table S4, Figures S4 and S5). For the P_top_Chip and P_top_WGS strategies, we found a trend that the prediction accuracy of the different prediction models increased slightly with the proportion of selected top SNPs. For the G_top_Chip and G_top_WGS strategies, we observed that the prediction accuracy of different prediction models was not robust and varied across traits and proportions of selected top SNPs. In many cases, this resulted in no improvement in prediction accuracy or even a decrease compared to using all markers from the SNP chip data.

**Figure 3:**
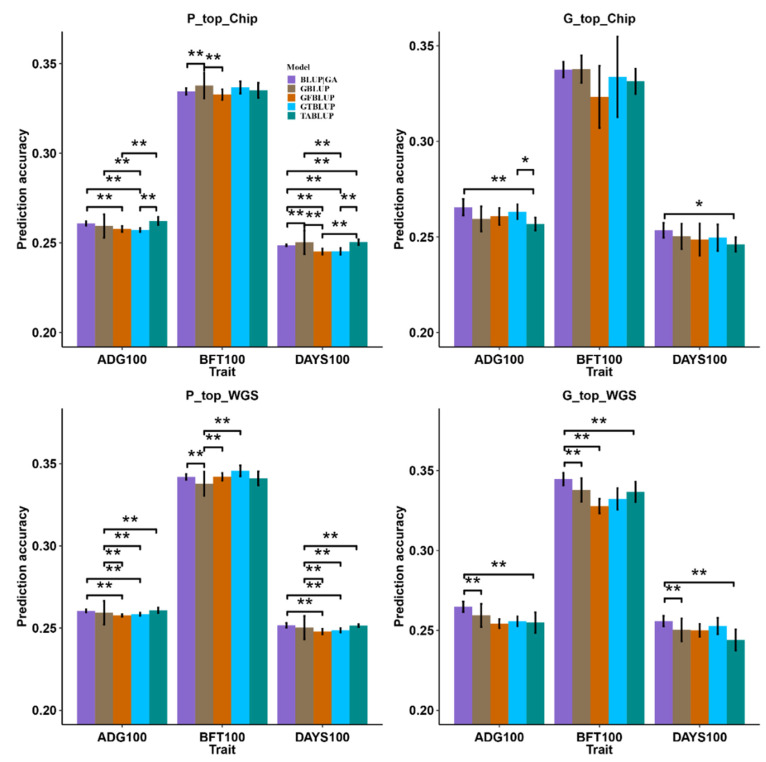
The prediction accuracy of different prediction models for growth and carcass traits with different SNP preselection strategies. “P_top_Chip” and “P_top_WGS “represent top SNPs based on *p*-value ranking from a large GWAS meta-analysis in the SNP chip and WGS data, respectively. “G_top_Chip” and “G_top_WGS” represent top SNPs based on estimated marker effects from an rr-BLUP analysis in the SNP chip and WGS data, respectively. * means *p* < 0.05, ** means *p* < 0.01. ADG100: average daily gain at 100 kg, BFT100: average back fat thickness at 100 kg, DAYS100: days to 100 kg.

To determine the statistical significance of different weighted models, we performed multiple paired t-tests based on the prediction accuracy of different strategies (Figure 3). For the P_top_Chip strategy, all the prediction models had very limited advantages over GBLUP (Figure 3). The prediction accuracy of the BLUP|GA and TABLUP models for ADG100 was slightly higher (0.15 to 0.28%) than GBLUP, but the difference did not reach significance. The prediction accuracy of the TABLUP model for ADG100 and DAYS100 was significantly higher (0.4 to 0.5%) than other prediction models. For the G_top_Chip strategy, the prediction accuracy of the BLUP|GA model for ADG100 was slightly higher (0.6%) than GBLUP, but the difference did not reach significance (Figure 3). The prediction accuracy of the BLUP|GA model for ADG100 and DAYS100 was significantly higher (0.7 to 0.9%) than the TABLUP model. In the context of the P_top_WGS strategy, the prediction accuracy of the BLUP|GA, GFBLUP, and GTBLUP models for BFT100 displayed significant advantages (0.4 to 0.8%) over the GBLUP model, as illustrated in Figure 3. The prediction accuracy of the TABLUP model for ADG100 and DAYS100 was significantly higher (0.1%) than GBLUP. In addition, the prediction accuracy of BLUP|GA for ADG100 and DAYS100 was significantly higher (0.2 to 0.4%) than the GFBLUP and GTBLUP models. For the G_top_WGS strategy, the prediction accuracy of the BLUP|GA model for three traits was significantly higher (0.1 to 1.0%) than the GBLUP and TABLUP models (Figure 3).

## 4. Discussion

The use of preselected variants based on the genetic architecture of complex traits as a strategy for genomic prediction offers an attractive approach to enhance predictive accuracy [11]. In pursuit of this objective, we compared the accuracy of genomic prediction based on the SNP chip data with that based on sets of preselected variants from a large GWAS meta-analysis. Our results indicated that the use of preselected variants from a large GWAS meta-analysis has shown small and non-robust improvements in the accuracy of genomic prediction compared to using all variants from the SNP chip data in pigs. A previous study [22] based on preselected variants by *p*-value ranking from GWAS results demonstrated an improvement in the accuracy of genomic prediction for BFT, ADG, and loin muscle area across different pig breeds. The improvement ranged from 4 to 13.2% compared to the SNP chip data, which was slightly higher than our results. Our observations might be attributed to several underlying factors. Firstly, the advantage of using preselected variants from a large GWAS meta-analysis may also be limited by the effective population size of the current selection population and the sizes of training sets. In a previous simulation study, it was discovered that the accuracy of genomic prediction was obviously lower in populations with a small effective population size across different population sizes [39]. Meanwhile, a previous study has shown that various strategies for preselected variants from WGS data based on GWAS results can improve the accuracy of genomic prediction, especially with larger training sets, when compared to using SNP chip data [21]. Secondly, the GWAS meta-analysis summary results are influenced by LD, which could introduce noise and limit the fine mapping of associated regions when using WGS data [21]. For example, in cases where a single-population GWAS fails to identify the causal variant, a meta-analysis may uncover another variant that exhibits greater significance than the causal variant itself [40]. This could be attributed to the presence of a stronger LD between these two variants within the same region. Finally, GWAS meta-analysis with WGS data can be affected by false positives in a more severe way than GWAS meta-analysis with SNP chip data, especially for highly polygenic traits [21]. In our study, the GWAS meta-analysis summary results were obtained using imputed WGS data, which may have introduced false positives due to limitations in sample size and the number of minor alleles contributing to an SNP across participating studies [41]. In addition, according to Zhang et al. [30], the use of preselected variants based on their size of effect estimated from an rr-BLUP model for genomic prediction improved the accuracy of genomic prediction (increased by 1.9 to 40%) compared to using all variants from WGS data. This strategy provides several advantages as it captures the genetic architecture of complex traits through the inclusion of SNPs with unknown effects, particularly in scenarios involving small data sets and/or traits with low heritability.

The predictive ability of preselected variants also depends on the proportion of selected top SNPs from the GWAS meta-analysis summary results. For this purpose, we compared the prediction accuracy using different proportions of selected top SNPs across different SNP preselection strategies. Among these strategies, namely the P_top_WGS and G_top_WGS strategies across all traits, a discernible pattern emerged, characterized by an initial increase in prediction accuracy followed by a subsequent decline. Notably, this trend was most pronounced when using the top 1% or 5% of selected SNPs, aligning with findings by Zhang et al. [30]. This is due to the occurrence of the inclusion of a larger number of trait-related SNPs in the model. However, as the number of available SNPs increased, the number of SNPs that were not correlated with the trait also increased. This, in turn, elevated the noise level for non-associated markers and caused a decline in prediction accuracy, approaching that observed when using all markers from the SNP chip data. In addition, Akbarzadeh et al. [42] considered that the number of SNP markers included in the prediction model is an essential factor for the prediction model.

Genomic prediction models need to be improved to leverage the potential of selected top SNPs from GWAS meta-analysis summary results. In this study, we compared the accuracy of genomic prediction of five models—GBLUP, GFBLUP, TABLUP, GA|BLUP, and GTBLUP models—among different SNP preselection strategies. The results showed that the superiority of BLUP|GA, which used a GBLUP with a weighted G matrix, was the largest when using a weighting factor of estimated marker effects from GWAS meta-analysis. For the P_top_Chip and P_top_GWAS strategies, the BLUP|GA model not only captured the large number of significant SNPs associated with traits from large-scale GWAS meta-analysis results but also efficiently utilized the estimated marker effect for constructing weighted G matrices. Many previous studies have reported that the BLUP|GA model leads to better genomic predictions than GBLUP, where weighting factors were obtained with estimated marker effects from either the GS model or GWAS model in the studied population [30,43]. A previous study demonstrated that a G matrix weighted with the estimated marker effect from the GWAS model may mitigate the adverse effects of imprecise estimated marker effects [29]. In GWAS results, it is common to observe an overestimation of QTL effects in the total estimated SNP effects [29]. This occurs due to the presence of several linked markers, all estimating the effect of the same QTL. Additionally, the total SNP variances often tend to be significantly larger than the total additive genetic variance. However, the issue of overestimation is no longer a problem when the estimated marker effect from a GWAS model is used as a weighting factor [28]. In addition, Ren et al. [28] reported that the estimated marker effect for constructing weighted G matrices from the GWAS method can significantly increase prediction accuracy compared with general GBLUP. Conversely, Su et al. [29] reported that the G matrix weighted with the estimated marker effect using the GWAS model resulted in lower accuracies than the general G matrix. Alternatively, the increased prediction accuracy in our study may be attributed to the accurate marker effect estimates obtained through the GWAS meta-analysis method.

For the G_top_Chip and G_top_WGS strategies, the BLUP|GA model captured the genetic architecture provided by the SNPs of larger effect from the rr-BLUP model. It also efficiently utilized the estimated marker effect from large-scale GWAS meta-analysis summary results to construct weighted G matrices. The findings indicated that, among the G_top_Chip and G_top_WGS strategies, the BLUP|GA model consistently exhibited the highest degree of robustness in terms of genomic prediction accuracy across all considered traits. This result is consistent with the results of similar studies by Zhang et al. [30], where using the genetic architecture provided by SNPs from the rr-BLUP model for genomic prediction resulted in a significant enhancement of the accuracy of predictions compared to GBLUP. This may occur because the genetic architecture part (S matrix) could effectively improve the prediction ability of the G matrix by capturing major genes affecting traits. In addition, the increase in accuracy is also possibly due to the increased similarity between the T matrix and the genetic relationship matrix at unobserved causal loci [30,44].

In our study, the superiority of the TABLUP model for ADG100 and DAYS100 outperformed other predictive models in the G_top_Chip and G_top_WGS strategies. For the G_top_Chip and G_top_WGS strategies, compared with the BLUP|GA model, the TABLUP model only utilized preselected SNPs based on estimated marker effects of rr-BLUP analysis for constructing $S_{a}$ matrices, as well as the estimated marker effect from the large-scale GWAS meta-analysis summary results for weighting factors. The superiority of the TABLUP model over the GBLUP and rrBLUP models has been investigated previously and is nearly equally to BayesB in terms of accuracy of genomic prediction [30]. In these models, the TABLUP model places greater emphasis on variants that explain a larger proportion of the genetic variance in the trait. In addition, the increased predictive accuracy of the TABLUP model may also be attributed to the fact that ADG100 and DAYS100 have complex genetic backgrounds and may be influenced by loci with both large and moderate effects simultaneously. However, the GTBLUP and GFBLUP models performed slightly lower compared to other models in our study, where the GTBLUP model has an advantage over the GFBLUP model in terms of prediction accuracy. This is probably due to the use of different genomic relationship matrices in the models. In contrast to the GFBLUP model, the GTBLUP model utilized the estimated marker effect from the large-scale GWAS meta-analysis summary results for weighting factors in the $S_{f}$ matrix. This is further evidence that utilizing the information provided by large-scale GWAS meta-analysis can increase prediction accuracy.

## 5. Conclusions

Our results show that the use of preselected variants based on a large-scale GWAS meta-analysis has potential to improve the accuracy of genomic prediction compared to all variants from the SNP chip data in pigs. However, the prediction accuracy using a given set of preselected variants exhibited a marginal improvement compared to all variants from the SNP chip data in pigs. By evaluating the effect of different proportion of selected top SNPs on genome prediction, we found that the accuracy of genomic prediction exhibited a trend of either initially increasing and then decreasing or simply decreasing across different SNP preselection strategies when the proportion of selected top SNPs increased. The BLUP|GA model, which incorporates SNP weights derived from posterior estimated marker effects obtained through a comprehensive GWAS meta-analysis, the potential to enhance predictive accuracy relative to the GBLUP model. The results of this study provide a strategy to maximize the advantage of using preselected variants based on large-scale GWAS meta-analysis, providing valuable insights for future applications of genomic prediction using extensive genotype data in livestock and poultry.

## Figures and Tables

**Table 1:** Summary statistics of growth and carcass traits in Large White pigs.

Traits ^1^	Number	Minimum	Maximum	Average	SD	CV
ADG100/g	1026	443	764	589	49	8
BFT100/mm	1026	3.82	18.8	9.522	2.257	23.702
DAYS100/d	1026	129.08	222.43	168.773	13.982	8.285

^1^ ADG100, average daily gain at 100 kg; BFT100, average back fat thickness at 100 kg; DAYS100, days to 100 kg; SD, standard deviation; CV, coefficient of variation.

**Table 2:** Number of selected top SNPs from the SNP chip data and the WGS data in the study population.

The Proportion of Selected Top SNPs	ADG100 ^1^		BFT100 ^1^		DAYS100 ^1^	
	Chip	WGS	Chip	WGS	Chip	WGS
0.1%	-	854	-	858	-	856
0.3%	-	2562	-	2574	-	2569
0.5%	-	4270	-	4290	-	4281
1%	382	8541	382	8580	383	8563
5%	1912	42,704	1908	42,898	191	42,814
10%	3824	85,409	3816	85,797	3833	85,629
20%	7648	170,818	7632	171,594	7666	171,257
40%	15,297	341,636	15,264	343,188	15,332	342,514
60%	22,945	512,453	22,897	514,781	22,999	513,771
80%	30,594	683,271	30,529	686,375	30,665	685,028
100%	38,242	854,089	38,161	857,969	38,332	856,285

^1.^ ADG100, average daily gain at 100 kg; BFT100, average back fat thickness at 100 kg; DAYS100, days to 100 kg; “Chip” represents all variants from the SNP chip data, and “WGS” represents all variants from the WGS data.

## Data Availability

Pig Genotype-Tissue Expression (PigGTEx) raw whole-genome sequence data and the GWAS meta-analyses summary statistics are available at http://piggtex.farmgtex.org/ (accessed on 1 April 2023) and http://pigbiobank.farmgtex.org (accessed on 1 April 2023), respectively. All other relevant data are available in the manuscript, supporting information files, and from the corresponding author upon request.

## Supplementary Materials

The following supporting information can be downloaded at: https://www.mdpi.com/article/10.3390/ani13243746/s1, Figure S1: the frequency distribution of the data of growth and carcass traits; Figure S2: the influences of the BLUP|GA model with weight parameter for genomic prediction using different proportion of selected top SNPs from the SNP chip data; Figure S3: the influences of the BLUP|GA model with weight parameter for genomic prediction using the proportion of selected top SNPs from the WGS data; Figure S4: the influences of different prediction models for genomic prediction using the proportion of selected top SNPs from the SNP chip data; Figure S5: the influences of different prediction models for genomic prediction using the proportion of selected top SNPs from the WGS data; Table S1: accuracy of the BLUP|GA model with weight parameter based on different SNP preselection strategies for growth and carcass traits in Large White pigs; Table S2: accuracy of using preselecting variants based on different SNP preselection strategies for growth and carcass traits in Large White pigs; Table S3: accuracy of the proportion of selected top SNPs based on different SNP preselection strategies for growth and carcass traits in Large White pigs; Table S4: accuracy of different prediction models based on different SNP preselection strategies for growth and carcass traits in Large White pigs.
