# Supplementary material for: Preselecting Variants from Large-Scale Genome-Wide Association Study Meta-Analyses Increases the Genomic Prediction Accuracy of Growth and Carcass Traits in Large White Pigs
Source: Animals (Basel). 2023 Dec 5;13(24):3746. doi: 10.3390/ani13243746 (PMC10740834; doi:10.3390/ani13243746)
Supplement: Supplementary file 1 [file animals-13-03746-s001.zip › Supplementary figure.pdf]

Supplementary figure

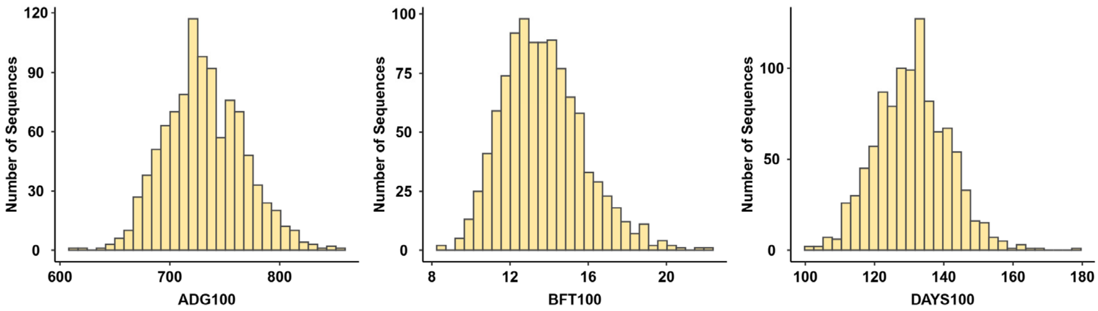

Figure S1 The frequency distribution of the data of growth and carcass traits ADG100: average daily gain at 100 kg, BFT100: average back fat thickness at 100 kg , DAYS100: days to 100 kg.

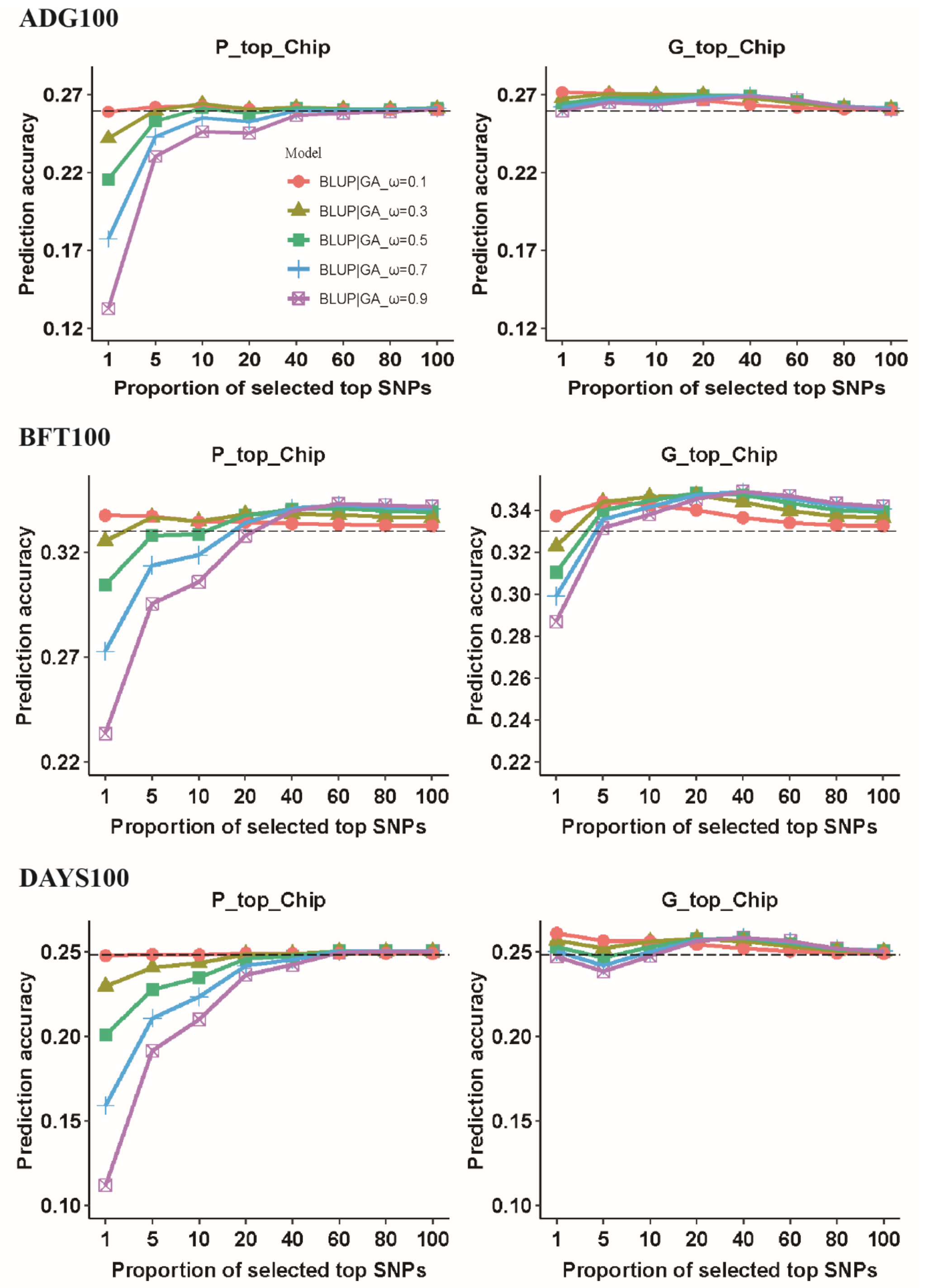

Figure S2 The influences of BLUP|GA model with weight parameter for genomic prediction using different proportion of selected top SNPs from the SNP chip data "P_top_Chip" represent top SNPs based on *P*-value ranking from a large GWAS meta-analysis in the SNP chip . "G_top_Chip" represent top SNPs based on estimated marker effects from an RR-BLUP analysis in the SNP chip. ADG100: average daily gain at 100 kg, BFT100: average back fat thickness at 100 kg , DAYS100: days to 100 kg. Dashed line at value of the SNP chip as a reference.

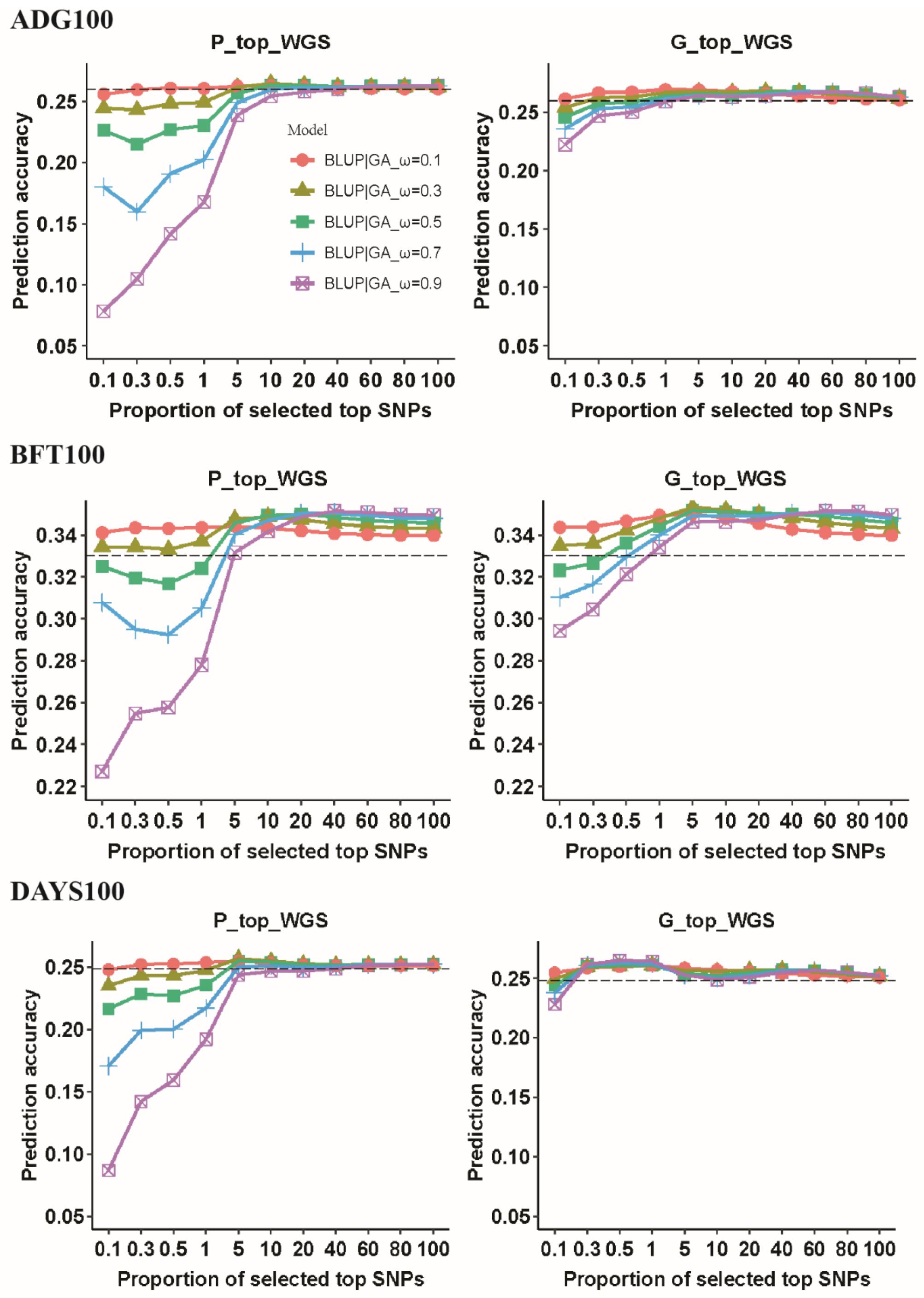

Figure S3 The influences of BLUP|GA model with weight parameter for genomic prediction using the proportion of selected top SNPs from the WGS data "P_top_WGS" represent top SNPs based on *P*-value ranking from a large GWAS meta-analysis in the WGS data. "G_top_WGS" represent top SNPs based on estimated marker effects from an RR-BLUP analysis in the WGS data. ADG100: average daily gain at 100 kg, BFT100: average back fat thickness at 100 kg , DAYS100: days to 100 kg. Dashed line at value of the SNP chip as a reference.

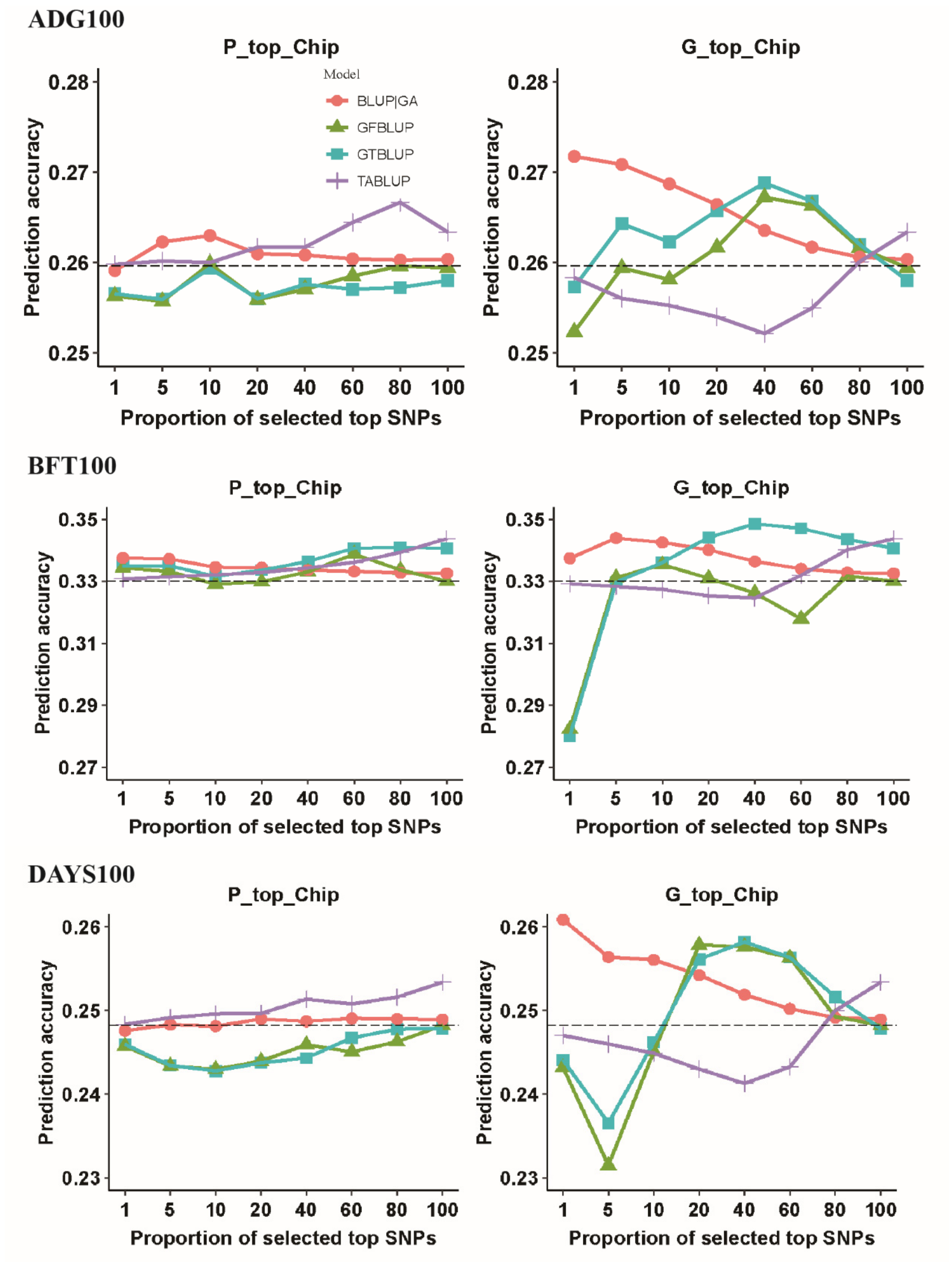

Figure S4 The influences of different prediction models for genomic prediction using the proportion of selected top SNPs from the SNP chip data "P_top_Chip" represent top SNPs based on *P*-value ranking from a large GWAS meta-analysis in the SNP chip . "G_top_Chip" represent top SNPs based on estimated marker effects from an RR-BLUP analysis in the SNP chip. ADG100: average daily gain at 100 kg, BFT100: average back fat thickness at 100 kg , DAYS100: days to 100 kg. Dashed line at value of the SNP chip as a reference.

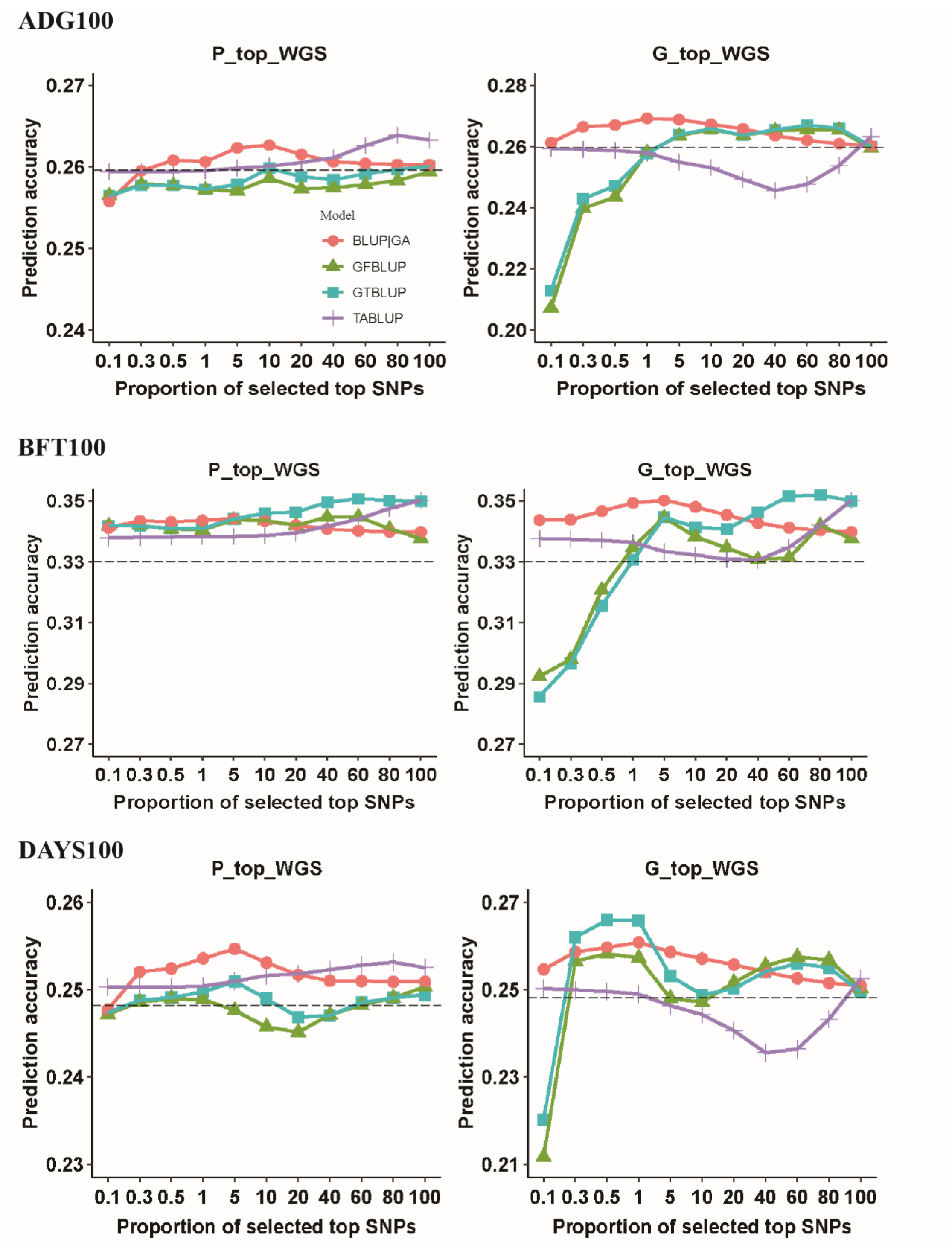

Figure S5 The influences of different prediction models for genomic prediction using the proportion of selected top SNPs from the WGS data "P_top_WGS" represent top SNPs based on *P*-value ranking from a large GWAS meta-analysis in the WGS data. "G_top_WGS" represent top SNPs based on estimated marker effects from an RR-BLUP analysis in the WGS data. ADG100: average daily gain at 100 kg, BFT100: average back fat thickness at 100 kg , DAYS100: days to 100 kg. Dashed line at value of the SNP chip as a reference.
